# Impact of Labile Zinc on Heart Function: From Physiology to Pathophysiology

**DOI:** 10.3390/ijms18112395

**Published:** 2017-11-12

**Authors:** Belma Turan, Erkan Tuncay

**Affiliations:** Department of Biophysics, Ankara University, Faculty of Medicine, 06100 Ankara, Turkey; erkan.tuncay@medicine.ankara.edu.tr

**Keywords:** zinc transporters, intracellular labile zinc, heart failure, endoplasmic reticulum stress, left ventricle

## Abstract

Zinc plays an important role in biological systems as bound and histochemically reactive labile Zn^2+^. Although Zn^2+^ concentration is in the nM range in cardiomyocytes at rest and increases dramatically under stimulation, very little is known about precise mechanisms controlling the intracellular distribution of Zn^2+^ and its variations during cardiac function. Recent studies are focused on molecular and cellular aspects of labile Zn^2+^ and its homeostasis in mammalian cells and growing evidence clarified the molecular mechanisms underlying Zn^2+^-diverse functions in the heart, leading to the discovery of novel physiological functions of labile Zn^2+^ in parallel to the discovery of subcellular localization of Zn^2+^-transporters in cardiomyocytes. Additionally, important experimental data suggest a central role of intracellular labile Zn^2+^ in excitation-contraction coupling in cardiomyocytes by shaping Ca^2+^ dynamics. Cellular labile Zn^2+^ is tightly regulated against its adverse effects through either Zn^2+^-transporters, Zn^2+^-binding molecules or Zn^2+^-sensors, and, therefore plays a critical role in cellular signaling pathways. The present review summarizes the current understanding of the physiological role of cellular labile Zn^2+^ distribution in cardiomyocytes and how a remodeling of cellular Zn^2+^-homeostasis can be important in proper cell function with Zn^2+^-transporters under hyperglycemia. We also emphasize the recent investigations on Zn^2+^-transporter functions from the standpoint of human heart health to diseases together with their clinical interest as target proteins in the heart under pathological condition, such as diabetes.

## 1. Introduction

Zinc is a redox inactive element and presents in almost all biological tissues. Zinc is accepted to be a component of antioxidant defense system and contributes to maintain the cell redox balance through different mechanisms [1]. There is a close relation between cellular labile zinc (Zn^2+^), endogenous processes, and biological macromolecules under physiological condition, mainly due to its unique property being their structural component or major regulator of macromolecules [2]. Labile Zn^2+^ plays an important role as a signaling molecule in large number of cells and tissues being a constituent of many proteins and enzymes in human body [3,4,5]. For this reason, even mild zinc-deficiency may impact numerous aspects of human health, including heart function [6]. Although Zn^2+^ has traditionally been regarded as relatively nontoxic, recent studies have shown how high intracellular labile Zn^2+^ level ([Zn^2+^]_i_) can be a potent killer of numerous cell types [7] including cardiomyocytes [8,9,10]. In cardiomyocytes, [Zn^2+^]_i_ is measured to be less than one nanomolar under physiological conditions being much less than the intracellular free Ca^2+^ ([Ca^2+^]_i_) [8,11]. Moreover, oxidants caused about 30-fold increase in [Zn^2+^]_i_ but only 2-fold in [Ca^2+^]_i_ in cardiomyocytes [8]. Therefore, any increase in [Zn^2+^]_i_ may be much more toxic biologically than is generally realized. Furthermore, any evolution of biomolecules to scavenge [Zn^2+^]_i_ is crucially important in ameliorating the cellular toxicity. Supporting these above statements, early studies showed that any disruption in [Zn^2+^]_i_ homeostasis could be associated with severe disorders, including injuries to cardiac tissues [6,12]. Another interesting action of [Zn^2+^]_i_ has been shown its insulino-mimetic activity in diabetic patients [13].

It has been shown that [Zn^2+^]_i_ in mammalian cardiomyocytes plays an important role in excitation-contraction coupling [8,9] and in excitation-transcription coupling [14]. In mammalian cells, [Zn^2+^]_i_ homeostasis is regulated with different ways. Mainly, cellular [Zn^2+^]_i_ is controlled via its pools. It can be released from metalloproteins (a structural component) or metalloenzymes (a cofactor) under pathological conditions. As up to 15% of the total cellular zinc can be bound to metallothioneins (MT), they can represent a significant pool of Zn^2+^ [15,16]. Other type storage for Zn^2+^ is intracellular compartments such as organelles and vesicles. Zn^2+^ transport is mediated by Zn^2+^-dependent proteins, named Zn^2+^ transporters, which are localized to both sarcolemma and intracellular membranes [17,18]. MTs and two Zn^2+^ transporter families, solute carrier 39A [SLC39A] or Zrt-, Irt-like proteins 7ZIPs and solute carrier 30A (SLC30A) or ZnTs play crucial roles to maintain the cellular Zn^2+^ homeostasis [19,20,21,22,23]. Although there are many recent very good review articles focused on recent progress to describe the physiological and biological functions of ZIP and ZnT transporters and provided a better understanding of Zn^2+^ biology, in here, we aimed to review the recent data on Zn^2+^ signaling by Zn^2+^ transporters in the heart under physiological and pathological conditions, and therefore, to update our current understanding on the role of cellular [Zn^2+^]_i_ regulation in heart function.

## 2. Role of Zinc in Human Health

Zinc, as one of important micronutrients in human nutrition, has very prominent role in the maintenance of tissue functions. As mentioned in a number of studies and review articles, its recognition for biological systems mostly depends on its being an essential component of many enzymes and its role in many physiological or metabolic processes in the mammalian body due to it being the most abundant intracellular metal ion found in cytosol, vesicles, organelles, and in the nucleus [2,3,4,7,24,25,26,27,28,29,30]. Therefore, trace element zinc has greatly attracted the attention of biological scientists for its importance in clinical medicine. Furthermore, its role in several nutritional disorders has been clearly established [2,31,32,33,34]. Although the demonstration of zinc essentiality for the growth of *Aspergillus niger* is very early in the literature [35], a recognition of its importance for the growth of plants and animals was not appreciated until the 1950–60s, what was considered improbable until 1960. It was believed that this element could be toxic if it was over its physiological level with high zinc intakes [36,37,38], although zinc was essential for human health and that its deficiency in humans would never occur [39].

Although Zn^2+^ is the most abundant intracellular metal ion found in cytosol, vesicles, organelles, and in the nucleus of mammalian cells including cardiomyocytes [11,40], even a small deficiency is a disaster to human health due to the number of biological dysfunctions [39]. In the literature, there are about 7000 articles searched using the key words “zinc deficiency and humans.” However, there is an early study on this topic published in *Nature* related with role of zinc deficiency in fruit trees in Britain by Bold C et al. [41]. Following this work, most studies were performed in animals, until the first observation in humans by Prasad et al. [39]. Important roles of Zn^2+^ associated with health implications and pharmacological targets have evoked further interest regarding its status in human health and nutrition.

Several review articles besides original works emphasized the central role of zinc content in human health starting from maternal period using documents from both underdeveloped and developing countries [27]. Zinc deficiency in humans seems to be widespread throughout the world. Generally, it is assumed that zinc deficiency in humans is due to inadequate dietary intake, gut malabsorption, or defective metabolism whereas at the cellular level, decrease zinc level can be caused a large series of morbid processes [5,8,29]. Recent clinical studies focusing on population-based groups demonstrated that there are conflicting results associated with zinc deficiency and related symptoms in different populations [42,43,44]. Moreover, some studies showed a positive correlation between serum zinc and type 2 diabetes risks in either middle-aged and older Finnish men or the Norwegian population as well as in development of liver fibrosis in the Miami adults [45,46,47,48]. Additionally, Kunutsor and Laukkanen [49] performed a population-based cohort study in the same country and, due to their data, they suggested that a higher serum zinc concentration is positively and independently associated with incident hypertension in men. However, clinical diagnosis of marginal zinc deficiency in humans remains problematic [50].

It is now well accepted that a well-controlled Zn^2+^ homeostasis via Zn^2+^ regulatory mechanisms can control influx/efflux of zinc to prevent toxic zinc accumulation into cells, particularly endogenous labile Zn^2+^ plays a significant role in cytotoxic events at single cell level, including cardiomyocytes [8,9,10,40,51]. As mentioned in many articles, Zn^2+^ is essential for growth and development for both plants and mammalians. At the cellular level, it is critically involved in proliferation, differentiation, and apoptosis [52,53,54,55,56]. Indeed, Zn^2+^ was known as relatively harmless comparison to several other metal ions with similar chemical properties, while there is not much interest to Zn^2+^ toxicity in biological systems although it has been known to be hazard in industrial aspects [36,57,58]. It is believed that overt symptoms of zinc toxicity require its large amount ingestion by the body. On the other hand, it has been known how zinc compounds as supplementary agents in daily diets are important for humans, particularly under some special conditions.

However, there are still widespread controversies and ambiguities with respect to the toxic effects and mechanisms of excess zinc in humans. Authors using different zinc-compounds, particularly metallic nanoparticles including zinc, demonstrated that the solubility and the size of zinc compounds have a major role in the induced toxic responses, whereas uptake of their large ones inside the cells was likely to play a key role in the detected cell cycle arrest [59]. In a recent interesting study demonstrated another important role of heavy metals including zinc in pathological biomineralization of cancer tissue due to their important roles in morphogenesis of tumors considering their ability to enter into covalent bonds with calcium salt molecules. Romanjuk et al. [60] examined the role of microelements in breast cancer calcifications. Their data demonstrated how excess heavy metals (such as iron, copper, chromium, and nickel) in the body could lead to pathologies in the tissues/organs, at most, via progressively increasing rate of degenerative/necrotic alterations. However, Hoang et al. [61] discussed in a widespread manner how zinc is an important metal as a possible preventive and therapeutic agent in pancreatic, prostate, and breast cancer in humans.

As summary of this part, the body zinc level in humans as well as zinc intake with nutrition, are receiving increasing attention, also due to its putative role in the development of different pathological conditions, there are serious conflicts between publications.

## 3. Labile Zn^2+^ in Cardiac Physiology and Pathology

As discussed in many review articles, zinc is a vital nutrient for human health via its incredibly important roles in physiology and pathology of many organ functions, at most, due to its important roles in proteins and enzymes [62,63,64]. As a consequence, zinc is required for the function of organs, including the heart. Furthermore, impairment of Zn^2+^ homeostasis is associated with a variety of health problems such as cardiovascular disorders [65,66,67,68]. Previously, several authors have documented the importance of zinc in cardiovascular function and diseases in many good review articles [1,66,69,70,71,72,73]. In these articles, it has been focused on the critical role of intracellular labile Zn^2+^ in the redox signaling pathway, where certain triggers lead to release of Zn^2+^ from proteins/intracellular pools and cause myocardial damage [8,14,40,51,74]. Thus, the area of Zn^2+^-homeostasis seems to be emerging in cardiovascular disease. However, it has been demonstrated that labile Zn^2+^ outside a narrow concentration range are toxic to a variety of cells [69], including cardiomyocytes [8,9,10,14,40,51,75].

It has been known, at cellular levels, that certain pathological conditions including hyperglycemia and/or changes in specific signaling pathways can stimulate the production of molecules related with the redox-state of the cells. Among these changes, the alterations in the voltage-dependent ionic channels, ion transporters and some ion exchanges [76]. Similar to [Ca^2+^]_i_ homeostasis, any alteration in [Zn^2+^]_i_-homeostasis, including redox-status of the cells under hyperglycemia, can be involved in development of cardiac dysfunction [8,9,77,78,79,80,81]. In that regard, it is clear that a well-controlled redox-status in cardiomyocytes can be very beneficial, in part, via mediation of either [Ca^2+^]_i_ homeostasis, [Zn^2+^]_i_ homeostasis or both for a cardioprotective approach in diabetic cardiomyopathy as well as other pathologies in patients with heart disease [51,82]. Therefore, it can be suggested that a well-controlled regulation of [Zn^2+^]_i_ homeostasis at cellular level, similar to [Ca^2+^]_i_ homeostasis, can have important strategy to protect heart against redox-unbalanced pathologies [12]. In this regard, in an early study by Kamalov et al. [83], it has been mentioned that [Zn^2+^]_i_/[Ca^2+^]_i_ ratio in cardiomyocytes and mitochondria have optimal ranges, having important roles to control the redox-status as well as oxidative stress status of cells. As shown in many articles, any enhancement of antioxidant defenses in cells are providing benefits against these pathological conditions, including the control of [Zn^2+^]_i_ associated control of [Ca^2+^]_i_ homeostasis, particularly, via RyR2 [40,51,82,84,85]. As summary, ours and others’ studies have documented that, at tissue level, [Zn^2+^]_i_-homeostasis in heart is impaired by different signaling mechanisms, including oxidative stress in the heart, and thereby, plays important role in the pathogenesis of myocardial injury. Our current understanding of the roles of [Zn^2+^]_i_ homeostasis and [Zn^2+^]_i_ signaling in human myocardial injury is yet limited under any pathological stimulus.

The importance and complexity of Zn^2+^ action has been presumed to parallel the degree of Ca^2+^’s participation in cellular processes [9,51,72,86]. At cellular level, Zn^2+^ homeostasis is regulated through Zn^2+^ transporters, Zn^2+^-binding molecules, and Zn^2+^ sensors. Interestingly, most of studies related with the role of intracellular labile Zn^2+^ in cell function are associated with its toxicity, most probably, due to its service as an important secondary messenger in various intracellular signal transduction pathways [79,87].

As mentioned, in previous paragraphs, the cellular toxicity of exogenous and a redox-inert labile Zn^2+^ is characterized generally by cellular responses such as mitochondrial dysfunction, elevated production of reactive oxygen species/reactive nitrogen species ROS/RNS, loss of signaling quiescence leading to apoptosis in cells, cell death and increased expression of adaptive and inflammatory genes [2,4,5,6,8,9,14,23,88]. Central to the molecular effects of Zn^2+^ are its interactions with cysteinyl-thiols of proteins, which alter their functionality by modulating their reactivity and participation in redox reactions, as well as its a cis-acting element that is the binding site for metal-responsive transcription factor-1 (MTF-1). When cytosolic labile Zn^2+^ is increased, it can lead to an increase in MTF-1 activity, which in turn leads to an increase in activation of *MTF-1* target genes [8,9,51,78,89].

Ongoing studies together with early studies demonstrated that both Zn^2+^ deficiency and excess are detrimental to cells, causing growth retardation, important metabolic disorders and, particularly, inducing an impaired excitation-contraction cycling in cardiomyocytes [8,9,14,90]. Although total cellular Zn^2+^ is about 200 µM, the cytosolic labile [Zn^2+^]_i_ in cardiomyocytes is less than 1 nM under physiological conditions [8,11], whereas it can increase either ~30-fold with acute oxidant exposure or ~2-fold under chronic hyperglycemia [8,9,51]. Therefore, in general, not only can Zn^2+^ deficiency be detrimental, causing depressed growth and serious metabolic disorders, but excess Zn^2+^ can also be toxic to many cells [8,70,91,92].

## 4. Role of Cellular Labile Zn^2+^ in Electrical Properties of Cardiomyocytes

It is well accepted that a controlled Ca^2+^ signaling is key essential mechanism for regular function of cardiomyocytes. As mentioned in Section 3, the intracellular Ca^2+^ accumulation induced by increased production of ROS can cause injury and dysfunction of cardiomyocytes [93,94,95].

The sarcoplasmic reticulum (SR) is a main intracellular Ca^2+^ store in cardiomyocytes and ryanodine receptors (RyR2s) on the membrane of the SR play a central role in modulating Ca^2+^ signaling. These SR Ca^2+^ release channels contain many cysteine residues in their regulatory domain and putative Ca^2+^ pore region. These residues are susceptible to modification by oxidants [94,96,97].

Supporting the previous data, Woodier and co-workers [98] demonstrated nicely how cytosolic Zn^2+^ can act as a high affinity activator of RyR2 while their experimental approach enabled the study of RyR2 function under tight control of the chemical environment. Importantly, it has been widely discussed later how Zn^2+^ at 1 nM concentration has an ability to directly activate RyR2, which have a much higher affinity for Zn^2+^ than Ca^2+^ (about three-fold) [99]. Moreover, their data provided important information on the role of Zn^2+^ on RyR2 modulatory function in the absence of Ca^2+^ and presented a paradigm shift in our general understanding RyR2 activation during excitation-contraction coupling. Therefore, the already known data together with these new data provided an important mechanistic explanation associated with [Zn^2+^]_i_ dishomeostasis and certain cardiomyopathies, including diabetic cardiomyopathy [51,82,98,99].

In our early study, we, for the time, have shown that the intracellular labile Zn^2+^ level, [Zn^2+^]_i_ in rabbit cardiomyocytes, is less than 1 nM in physiological conditions [8], about 100-fold less than [Ca^2+^]_i_. At various concentrations, labile Zn^2+^ leads to the release of toxic ROS [79]. More importantly, our data demonstrated that [Zn^2+^]_i_ was increased markedly (about 30-fold but only doubled [Ca^2+^]_i_) with thiol-reactive oxidants and contributed to oxidant-induced alterations of excitation-contraction coupling ECC under in vitro conditions. Therefore, that information emphasized the importance of [Zn^2+^]_i_ measurement such as, how it could lead to significant overestimation of [Ca^2+^]_i_, if it was overlooked [8]. In later studies, under in vivo conditions, [Zn^2+^]_i_ was increased by 70% in diabetes [51,78] and over 200% in aldosteronism [100]. Interestingly, Xie and Zhu [101] aimed to understand better the modulation of RyR2s during oxidative stress and showed how RyR2 in rat ventricular myocytes was modulated biphasically by sulfhydryl oxidation, contribution of increased [Zn^2+^]_i_ besides increased [Ca^2+^]_i_.

Although it has been shown the resting level of [Zn^2+^]_i_ in cardiomyocytes, there was very little information about precise mechanisms controlling intracellular distribution of Zn^2+^ and its variations during cardiac function. Therefore, we aimed to investigate the rapid changes in Zn^2+^ homeostasis in detailed and using the Zn^2+^-specific fluorescent dye, FluoZin-3, in comparison to Ca^2+^-dependent Fluo-3 fluorescence, we, for the first time, vizualised the existence of Zn^2+^ sparks and Zn^2+^ transients, in quiescent and electrically-stimulated cardiomyocytes, similarly to known rapid Ca^2+^ changes, while both Zn^2+^ sparks and Zn^2+^ transients required Ca^2+^ entry [9]. Inhibiting the SR-Ca^2+^ release, or increasing the Ca^2+^ load in a low-Na^+^ solution, suppressed or increased Zn^2+^ movements, respectively. Moreover, oxidation by H_2_O_2_ facilitated, and acidic pH inhibited the Ca^2+^-dependent Zn^2+^ release in freshy isolated rat ventricular cardiomyocytes.

**Figure 1:**
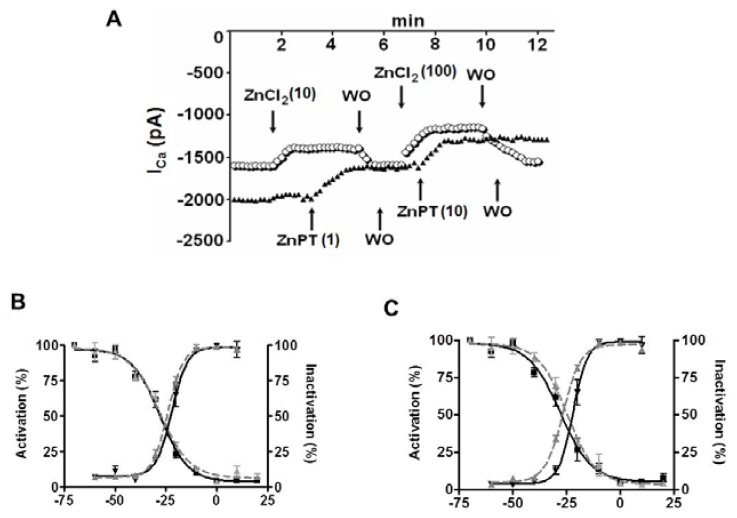
A patch-clamp study on the time course of L-type Ca^2+^-current (I_Ca_), depressed by Zn^2+^ exposed either extracellulary (ZnCl_2_) or intracellularly (i.e., loaded with Zn^2+^-ionophore pyrithione, ZnPT). (**A**) The I_Ca_ was evoked at 0 mV from a holding potential −80 mV and I_Ca_ was recorded in the presence of either ZnCl_2_ (10 and 100 µM) or ZnPT (1 and 10 µM) in whole-cell patch-clamped ventricular cardiomyocytes in the presence of 1.8 mM external Ca^2+^. WO represents the washout of applications. Corresponding availability curves of the I_Ca_ by either ZnCl_2_ (**B**) or ZnPT (**C**). Note about 10 mV left-shift by ZnPT application in availability curve of I_Ca_.

In historical background, studies show that most experimental studies on the role of [Zn^2+^]_i_ were performed in nervous system, at most, due to the localization of Zn^2+^ in nerve terminals and synaptic vesicles of excitatory neurons in the central nervous system [102,103]. The [Zn^2+^]_i_ homeostasis in mammalian cells results from a coordinated regulation by different proteins involved in the uptake, excretion, and intracellular storage/trafficking of Zn^2+^ [104]. It has been shown the Zn^2+^ influx via the L-type Ca^2+^ channels in heart cells [8,14,75] while it was via L-type and N-type Ca^2+^ channels in neurons [105]. As can be seen in Figure 1, here, we reinvestigated the effects of extracellular and intracellular Zn^2+^ on the L-type Ca^2+^ current. In the presence of external Ca^2+^, the L-type Ca^2+^ current is inhibited by external Zn^2+^ (ZnCl_2_) and intracellular Zn^2+^ loading (with Zn^2+^-pyrithione, ZnPT) also reduces the L-type Ca^2+^ current as a concentration- and time-dependent manner (Figure 1A). Although both effects are washable, the intracellular Zn^2+^ loading induced a marked leftward shift in inactivation of the channels (Figure 1C) without any effect under external Zn^2+^ exposure (Figure 1B). Similar to ours, Alvarez-Collazo et al. [106] demonstrated the modulation of transmembrane Ca^2+^ movements and their regulation by β-adrenergic stimulation with both basal intracellular and extracellular Zn^2+^, emphasizing the importance of well-controlled cellular [Zn^2+^]_i_-homeostasis for prevention/treatment of cardiac dysfunction, whereas Traynelis et al. [107] showed that both T-type Ca^2+^ current and L-type Ca^2+^ current are inhibited by excess external Zn^2+^ via inhibition of N-methyl-D-aspartate (NMDA) receptors. Although the exact underlying mechanisms of Zn^2+^ effects are not clear yet, surface charge effects could be invoked to explain some of the Zn^2+^ actions. However, as for other divalent metal cations, most of the effects of Zn^2+^ could be well explained by changes in the gating of ion channels [108,109,110] that we have shown in Figure 1 similar to our previous data [75]. Furthermore, squid K^+^ channels are far more sensitive to Zn^2+^ than Na^+^ channels but the interactions of Zn^2+^ with gating charges appear similar in both cases [109]. In that regard, Aras [111] reported that, during sublethal ischemia, the early rise in neuronal Zn^2+^, preceding the rise in intracellular Ca^2+^, was responsible for the hyperpolarizing shift in the voltage dependency of the delayed rectifier Kv2.1 channels.

Labile Zn^2+^, with even picomolar concentrations, modulates many cellular processes via either inhibiting or activating many proteins, enzymes, kinases and phosphatases, particularly, at Ser/Thr or Tyr sites [2,29,112,113,114]. Therefore, these findings indicate clearly that cellular labile Zn^2+^ level has critical importance for cellular physiological function besides its physiopathological or toxicological role, and strongly suggest its modulatory activity of signal transduction processes [106,115]. Furthermore, it is known that fluctuations of [Zn^2+^]_i_ participate in important cellular functions of not only breast cancer cells [116] but also mammalian cardiomyocyte [51,82,89]. In diabetic rat heart, an important actor in contractile machinery family, RyR2 and its accessory kinases protein kinase A (PKA) and calmodulin-dependent protein kinase II (CaMKII) are phosphorylated, significantly, at most, due to increased oxidative stress via increased [Zn^2+^]_i_. Interestingly, these changes could be prevented with antioxidant treatment under either in vivo or in vitro conditions. Indeed, either excess Zn^2+^ exposure to cardiomyocytes or labile Zn^2+^ loading of cardiomyocytes with zinc-ionophore could induce marked phoshorylation in RyR2, PKA and CaMKII as well as transcription factors such as nuclear factor κB (NFκB) and glycogen synthase kinase-3 (GSK) and other endogenous actors such as protein kinase B also known as Akt [89]. Parallel to our data, it has been shown that labile Zn^2+^ inhibits the activity of adenylyl cyclases as well as the hormone and forskolin stimulation of cAMP synthesis in N18TG2 cells [117], and, by preventing guanosine-5'-triphosphate (GTP) binding to the GTPase [118]. Also, in the presence of Ca^2+^ and calmodulin, increasing concentrations (in micromolars) of Zn^2+^ caused a progressive inhibition of substrate phosphorylation by CaMKII such as to produce a concentration-dependent inhibition of phospholamban phosphorylation [119]. However, Yi and coworkers [120] examined the role of extracellular Zn^2+^ exposure on cardiomyocyte contraction-relaxation function by using molecular and cellular techniques and showed that RyR2 and phospholamban were markedly dephosphorylated after permeating the hearts with 50 μM Zn^2+^. The different results associated with RyR2 phosphorylation level with Zn^2+^-exposures, most probably depending on the differences between Zn^2+^-exposure periods. One group experiments were performed in hours, while others in minutes.

Recently, since M-type (Kv7, *KCNQ*) potassium channels are important for the control of the excitability of neurons and muscle cells, Gao et al. [121] studied the effect of intracellular labile Zn^2+^ on M-type (Kv7, KCNQ) K^+^-channels. Their results reported that [Zn^2+^]_i_ directly and reversibly augments the activity of recombinant and native M channels, being mechanistically distinct from the known redox-dependent KCNQ channel potentiation.

Taken into consideration the facts of [Zn^2+^]_i_ in many cell types such as operation of many physiological and pathological mechanisms of cell excitation via the suppression of activity or expression of ion-channels, transporters, pumps, and receptors or pharmacological augmentation of their activities as a recognized strategy for the treatment of hyper-excitability disorders, it would be very helpful to understand well the action of [Zn^2+^]_i_ in cardiomyocytes. However, physiological mechanisms resulting in ionic channel potentiation are rare. As short due to already known data, a large amount of Zn^2+^-proteins that are modulated by or contain Zn^2+^ can directly or indirectly affect the many cellular processes, at most, due to labile Zn^2+^ action on cell redox-balance [9,76]. At the cellular level, the effects of labile Zn^2+^ in cardiomyocytes via its action in membrane receptors, transporters and ionic channels as well as endogenous accessory proteins of contractile machinery and some transcription factors are summarized in Table 1 and Table 2, respectively.

## 5. Labile Zn^2+^ Pools in Cardiomyocytes

As described in previous sections, labile Zn^2+^ regulates the expression and activation of biological molecules such as ion channels, transcription factors, enzymes, adapters, and growth factors, along with their receptors in many cell types, including cardiomyocytes. Excess Zn^2+^ can be detrimental to cells, particularly that of cardiomyocytes [8,9,10,14]. As an intracellular signal transducer in multiple cellular functions, it has been shown that intracellular labile Zn^2+^ has an important role in ECC in cardiomyocytes by shaping Ca^2+^ dynamics [9,51,98], while it acts as a neuromodulator in synaptic transmissions [122,123]. In the regulation of cellular labile Zn^2+^, subcellular pools as well as metalloproteins are important actors, which also include manyZn^2+^-transporters [29,87,124].

In our previous study performed with freshly isolated ventricular cardiomyocytes, we demonstrated that rapid changes in labile Zn^2+^ mostly resulted from Zn^2+^ displacement by Ca^2+^ ions from intracellular binding sites that were highly sensitive to the redox status of the cardiomyocytes by using Zn^2+^-specific fluorescence dye, FluoZin-3 [9]. In order to examine the physiological importance of the protein-bound Zn^2+^ pools, similar to other studies [125] by applying acidic pH to the cardiomyocytes or by causing an oxidative stress with H_2_O_2_, we induced chemical modifications of the thiol groups in the proteins and demonstrated that Zn^2+^ binding to metallothioneins decreased at acid pH and significantly reduced contraction without altering [Ca^2+^]_i_.

**Figure 2:**
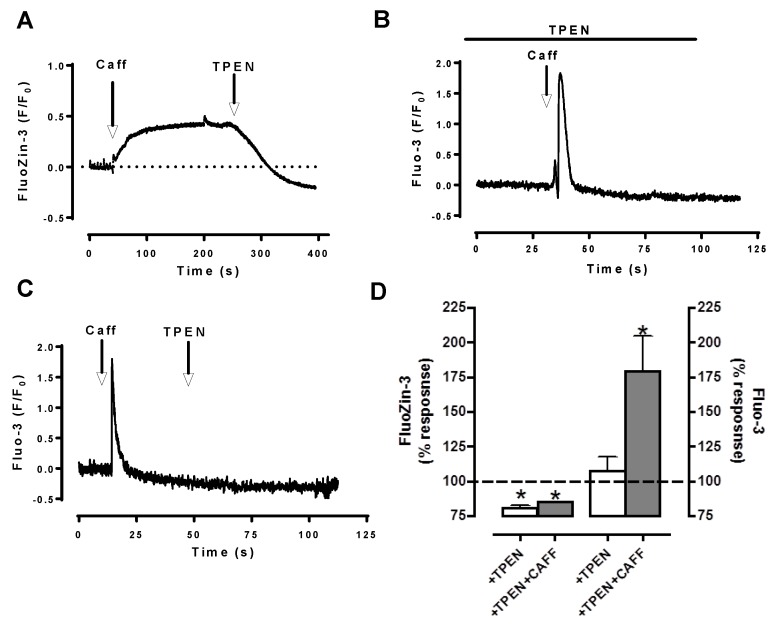
FluoZin-3 and Fluo-3 responses to caffeine application demonstrate sarco(endo)plasmic reticulum as an intracellular labile Zn^2+^ pool in cardiomyocytes performed with confocal imaging. We used either a Zn^2+^-specific fluorescence dye, FluoZin-3 (**A**) or a Ca^2+^-specific fluorescence dye, Fluo-3 (**B**) loaded ventricular cardiomyocytes isolated from rat heart and stimulated them with 10 mM caffeine (Caff) in the either absence (**A** and **B**, respectively) or presence of a membrane-permeant Zn^2+^ chelator TPEN (*N*,*N*,*N′*,*N′*-tetrakis(2-pyridylmethyl) ethylenediamine, 30-µM) (**C**), respectively. Normalized caffeine responses are given as F/F0, where F is the fluorescence signal and F_0_ is the diastolic fluorescence. The mean (± standard error of mean (SEM)) values are given for the protocols in (**D**), *n* = 5–6 for hearts/protocol. * *p* < 0.05 vs. before application.

Our testing S(E)R as an possible intracellular Zn^2+^ pool by using ryanodine application, we observed significantly and simultaneously decreases in the intensities of both Zn^2+^ transients and Ca^2+^ transients in field-stimulated cardiomyocytes without affecting their basal levels [9]. Furthermore, we performed additional experiments with caffeine. As can be seen in Figure 2, we observed two different responses in Fluo-3 or FluoZin-3 loaded cells. In Fluo-3 loaded cells, there was fast transitory and short-lived large increase as response to caffeine application, whereas there was a slow initial large increase followed with a stable long-lived plateau in FluoZin-3 loaded cells. These data support the hypothesis of sarco(endo)plasmic reticulum, S(E)R could be a Zn^2+^ pool similar to Ca^2+^ in cardiomyocytes. Furthermore, recently by using Förster resonance energy transfer (FRET)-based recombinant-targeted Zn^2+^-probes [11], we have shown that [Zn^2+^]_i_ in cardiomyocytes is calculated less than 1 nM, while ~5-fold higher in S(E)R, and less than cytosolic-level in mitochondria. Elevated cytosolic Zn^2+^ appears to contribute to deleterious changes in many cellular signaling-pathways including hyperglycemia-challenged cardiomyocytes [8,9,10,19,51]. Moreover, we also demonstrated that elevated cytosolic Zn^2+^ appears to be associated with loss of S(E)R Zn^2+^ via hyperphosphorylation of Zn^2+^-transporter ZIP7, which further induces endoplasmic reticulum (ER) stress in cardiomyocytes in the diabetic rat heart [40]. Of note, in eukaryotes, Ellis et al. [126] demonstrated the Zn^2+^-deficiency associated disruption in ER such as alteration in its function and induction of ER stress.

Additional studies also pointed out mitochondria to be another intracellular Zn^2+^-pool in cardiomyocytes [9]. Expose of FluoZin-3 loaded cardiomyocytes either to a mitochondrial complex I inhibitor or to carbonyl cyanide 4-(trifluoromethoxy) phenylhydrazone (FCCP) a mitochondrial protonophore induced rapid and significant inhibitory effects on the Zn^2+^-changes with only mild initial effects on the Fluo-3 loaded cells. In this regard, in cortical neurons, it was proposed that the source of Ca^2+^-dependent Zn^2+^ release appears largely to be mitochondria [127]. In cardiomyocytes, since mitochondria constitute the major source of intracellular ROS production [128], mitochondria-related excessive ROS production has been implicated in the pathogenesis of many cardiovascular diseases. Indeed, the Ca^2+^-dependency of the glutamate mobilization of intracellular Zn^2+^ in neurons is attributed to the generation of ROS arising from both cytosolic and mitochondrial sources [129]. In cardiomyocytes, Ca^2+^ influx might trigger transitory change in ROS production leading to Zn^2+^ transients even on this time scale. Such a hypothesis could account for our recent observations [40].

Besides voltage-dependent ionic channels, transient receptor potential (TRP) channels are a large family associated with multi-signal transducers and play important roles in different organ function, including heart function. The functional and structural control of TRP channels by trace metal ions, including Zn^2+^, has been demonstrated in different cell types [130]. For example, the high expression levels of both transient receptor potential cation channel3(TRPC3) and TRPC6 have been shown in the heart and could participate in the pathogenesis of cardiac hypertrophy and heart failure as a pathological response to chronic mechanical stress [131]. Additionally, the activation of transient receptor potential cation channel subfamily M member 4 (TRPM4), a Ca^2+^-activated, but Ca^2+^-impermeable non-selective cation channel, has been also demonstrated to have role in conduction block and other arrhythmic propensities associated with cardiac remodeling and injury [132]. Furthermore, Uchida and coworkers [133] have documented that extracellular Zn^2+^ regulates TRPM5 channel activation [133]. On the other hand, Lambert and coworkers demonstrated that extracellular Zn^2+^ exposure to HEK293 cells did inhibit TRPM5 and TRPM1 activity [134]. Moreover, Abiria and coworkers [135] recently showed that the majority of TRPM7 is localized in abundant intracellular vesicles in HEK293 cells and ROS-mediated TRPM7 activation releases Zn^2+^ from these vesicles following Zn^2+^ overload. They emphasized the important role of TRPM7-mediated Zn^2+^ release and the regulation of ROS signaling processes during postnatal stress/injury. Therefore, one can emphasized how will be very important to understand the roles of TRP channels as detailed in the regard of their contribution to the key procedures for the development of cardiovascular disorders. Furthermore, it seems that they may provide basic scientific knowledge for the development of new preventive and therapeutic approaches to manage patients with cardiovascular diseases [136].

**Figure 3:**
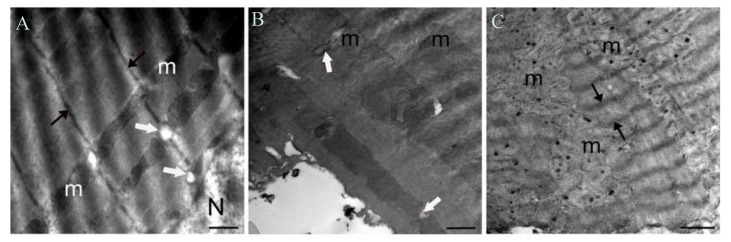
An electron microscopy examination of the effect of increased intracellular labile Zn^2+^ on ultrastructure of cardiomyocytes. The microscopic examination of freshly isolated cardiomyocytes under physiological condition (**A**) showing regular myofibrils and mitochondrial (m) structure and under 0.1 μM ZnPT incubation for 15–20 min (**B**) showing irregular mitochondrial cristae (white arrows). Magnification is ×21, 560 and bars represent 500 nm. On the right, cardiomyocytes showing clustered and degenerated mitochondria (m) under 1 μM ZnPT incubation for 15–20 min(C). Black arrows are showing irregular Z-lines. Magnification is ×12,930 and bar represents 1000 nm.

On this basis, the effects of increased intracellular labile Zn^2+^ on the structure of cardiomyocytes particularly focused on ultrastructure of mitochondria were examined by electron microscopy by using short-term ZnPT incubation (0.1 or 1 μM for 15–20 min) in freshly isolated ventricular cells. As can be seen in Figure 3A–C, marked irregular mitochondrial cristae and significantly clustered and degenerated mitochondria between the myofibrils together with electron-dense matrix were observed in labile Zn^2+^ loaded cells. Additionally, there were an important amount of fragmented mitochondria and rounding and swelling in mitochondrion. Although the sarcomere showed normal structural appearance with regular myofibrils and mitochondrial structure in the control group cells (Figure 3A), there were dramatic signs of injury in the form of condensation, increased matrix density, and deposits of electron-dense material in the loaded cardiomyocytes. These Zn^2+^ loading effects in mitochondria can support its Zn^2+^ sensing pool in cardiomyocytes. However, the early data by Jang et al. [137] showed that NO mobilizes intracellular Zn^2+^ via Cyclic guanosine monophosphate/Protein Kinase G (cGMP/PKG) signaling pathway and prevents mitochondrial oxidant damage in cardiomyocytes.

## 6. Zn^2+^ Transporters in Cardiomyocytes

Recent review articles well summarized the already published data, performed in different mammalian cells except cardiomyocytes, which showed the regulation of Zn^2+^ homeostasis via a number of Zn^2+^-transporters and how they are crucial for proper cellular functions [22,23,29,138,139,140,141,142]. In the review by Kambe [143], it has been pointed out how the impaired Zn^2+^ transporter functions into and out of cells strongly linked to clinical human diseases [144]. Since the membrane transporters having the great potential for drug targets [145,146], hence, cytosolic labile Zn^2+^ and Zn^2+^ transporters should be considered as novel therapeutic targets for diseases, including heart diseases. In this section, we aimed to describe the physiological and molecular functions of Zn^2+^-transporters, which regulate [Zn^2+^]_i_ homeostasis and are involved in signal transduction and heart diseases, particularly such as diabetes.

Even in early reviews, tightly control of [Zn^2+^]_i_ homeostasis is defined due to existence of specific Zn^2+^-transporters, including the coordinated regulation of [Zn^2+^]_i_ homeostasis in terms of its uptake, efflux, distribution, and storage, which are documented in several review articles, nicely [20,147]. In general, Zn^2+^ transporters belong to a family of transmembrane proteins that control the flux of Zn^2+^ across cellular membranes into cytosol (ZIPs) and out of cytosol (ZnTs) in many types of cells, therefore, contribute to the distribution, storage, and compartmentalization of Zn^2+^. Additionally, these all predict proteins with multiple membrane spanning regions, and most have a histidine-rich intracellular loop. The first described Zn^2+^ transporter in mammalian cells is ZnT-1 in kidney cells [148] while the second one is ZnT3 in regions of the brain [149]. The ZnT proteins (solute-linked carrier 30, SLC30) and the ZIP (zinc-regulated trans- porte /iron-regulated transporter Zrt/Irt)-like, solute-linked carrier, 39, *SLC39*) have been identified in mammalian tissues [22,23,150]. In mammals, there are 10 members of the Zn^2+^ efflux transporters (ZnT1–10) and 14 members of the zinc influx transporters (ZIP1–14).

ZIP proteins are thought to form homodimers to transport Zn^2+^ across the cellular membrane [151], while the conserved hydrophilic residue seems to sense metal specificity [152]. Supporting this statement, it has been demonstrated that ZIP8 and ZIP14, possessing glutamic acid instead of the conserved histidine, can efficiently transport Fe^2+^, Mn^2+^, and Cd^2+^ in addition to Zn^2+^. Human genetic disorders caused by mutations and single-nucleotide polymorphisms in Zn^2+^ transporter genes were summarized by Kambe et al. [144,153]. They documented that a number of genetic disorders are caused by mutations in the genes encoding ZIPs and ZnTs, such as ZIP4 in acrodermatitis enteropathica, ZIP13 in the spondylocheiro dysplastic form of Ehlers-Danlos syndrome, ZnT2 in transient neonatal zinc-deficiency, ZnT8 in type 1 and 2 diabetes mellitus, and ZnT10 in Parkinsonism and dystonia [22,23,141,142].

Furthermore, in the recent review article by Hara et al. [29], mechanisms of Zn^2+^-transporter expression and modification have been documented, very widely in different mammalian cells/tissues, focusing on their physiological roles from their molecular basis to genetic importance. In that review, they presented the role of ZnTs such as ZnT2, ZnT3, ZnT4, and ZnT8, which localize to acidic compartments and to vesicles such as endosomes/lysosomes, synaptic vesicles, and insulin granules as well as the ZIPs such as ZIP4, ZIP5, ZIP6, ZIP7, ZIP8, ZIP10, ZIP12, ZIP13, and ZIP14 in different cells. A number of cellular proteins, enzymes, kinases and phosphatases interact with Zn^2+^ for their biological functions. Studies have revealed that Zn^2+^ acts not only as an accessory molecule for proteins but also as a signaling molecule, much like Ca^2+^ [15,29].

Among others, ZnT7 has been demonstrated to play an important role in both growth and the accumulation of body fat in mice [154] as well as the association between its deficiency and metabolic disorders such as insulin and glucose intolerance and hyperglycemia [155]. Furthermore, studies on ZnT8 showed that the ZnT8 is strongly related to type 1 and 2 diabetes [156,157]. In this regard, later studies emphasized that ZnT8 expressed in pancreatic β-cells is involved in secreting insulin, forming crystals [158,159,160], and eliminating insulin by the liver [161]. In this field, the recent review articles by Chabosseau and Rutter [162] and by Rutter et al. [163] reviewed the regulation and roles of Zn^2+^ in islet cells and the mechanisms by which ZnT8 variants might affect glucose homeostasis and diabetes risk. Correspondingly, they presented that genetic variants in the ZnT8 gene, which encodes the diabetes-associated granule-resident ZnT8, are associated with an altered risk of type 2 diabetes. Additionally, they discussed the effects on insulin secretion and action of deleting or over-expressing ZnT8 highly selectively in the pancreatic β-cell, and the role of Zn^2+^ in insulin signaling. Due to their own data together with the others’ data, it has been concluded that maintenance of glucose homeostasis, and therefore lower diabetes risk, due to a proper intake level of dietary zinc at systemic level and a well-controlled [Zn^2+^]_i_ homeostasis at cellular level is provided with both insulin release and insulin action at physiological levels.

In the content of ZIPs’ roles, there are a limited number of studies in mammalian cells in the literature without any in cardiomyocytes except our study [40]. However, Ellis et al. [126] demonstrated that the zinc deficiency in ER leads to an unfolded protein response (UPR) in human cells. In a later study, Huang et al. [155] proposed that ZIP7 is localized to Golgi apparatus in Chinese-Hamster Ovary-cells, allowing Zn^2+^-release from Golgi lumen into cytosol. It has been also suggested that ZIP7 facilitates release of Zn^2+^ from ER [164] and behaves as a critical component in sub-cellular re-distribution of Zn^2+^ in other systems [165]. Additionally, it has been hypothesized that protein kinase-2 (CK2) triggers cytosolic Zn^2+^-signaling-pathways by phosphorylating ZIP7 [116], while some studies have also highlighted its important contribution to Zn^2+^-homeostasis under pathological conditions [166,167,168]. In addition, in a recent study, it was demonstrated that ZIP7, which predominantly localizes to the ER membrane, promotes rapid cell proliferation in intestinal crypts by maintaining ER function. They also found that mice with an intestinalepithelium-specific ZIP7 deletion exhibited extensive apoptosis in the stem-cell-derived transit-amplifying cells due to increased ER stress. They further showed that UPR signaling upregulates ZIP7, which maintains [Zn^2+^]_i_ homeostasis under ER stress and facilitates epithelial proliferation. Therefore, ZIP7 is considered as a novel regulator of [Zn^2+^]_i_ homeostasis of the intestinal epithelium [169].

Although studies have shown the presence of weakly expressed ZIP7 and ZnT7 in mammalian heart [170,171], their subcellular localizations and functional roles in cardiomyocytes were not yet known well. In that regard, we hypothesized that disruption of Zn^2+^-transporters and Zn^2+^-axis such as ZIP7 and ZnT7 might contribute to deleterious changes in diabetic cardiomyocytes. Therefore, we first clarified their subcellular localizations into S(E)R and then explored their functional roles in Zn^2+^ homeostasis, particularly under hyperglycemia. Additionally, we tested their roles in cytosolic Zn^2+^ re-distribution and development of ER-stress in hyperglycemic conditions, at most due to activation of casein kinase 2 alpha (CK2α) [40]. We observed markedly increased mRNA and protein levels of ZIP7 in ventricular cardiomyocytes from diabetic rats or high glucose-treated H9c2 cells whilst ZnT7 expression was low comparison to those of controls. Additionally, we observed increased ZIP7 phosphorylation in response to high glucose in vivo and in vitro in ventricular cardiomyocytes. Using recombinant targeted FRET-based sensors, we showed that hyperglycemia induced a marked redistribution of cellular labile Zn^2+^, increasing cytosolic labile Zn^2+^ and lowering labile Zn^2+^ in the S(E)R. These changes involve alterations in ZIP7-phosphorylation and were suppressed by siRNA-mediated silencing of CK2α. Due to our whole data, we, for the first time, demonstrated that opposing changes in the expression of ZIP7 and ZnT7 observed in hyperglycemia is very important for development of ER stress in the heart. In addition, we also pointed out an importance of sub-cellular labile Zn^2+^ re-distribution in the hyperglycemic heart, which is resulting from altered ZIP7 and ZnT7 activity and contributing to cardiac dysfunction in diabetes [40]. Furthermore, Myers [140] previously discussed very widely the roles of Zn^2+^ transporters and Zn^2+^ signaling by using recent new roles of Zn^2+^ and its transporters in the synthesis, secretion, and action of insulin are dependent on zinc and the transporters in type 2 diabetes. Author, particularly, emphasized the role of cellular Zn^2+^’s dynamic as a “intracellular second messenger” to control insulin signaling and glucose homeostasis. Therefore, it was raised extensively a new research field into the pathophysiology of insulin resistance and possibility of new this-field related drug targets in diabetes [13,172,173,174,175].

In conclusion, the already known data associated with the role of ZIPs and ZnTs provide novel insights into regulation of cellular-Zn^2+^ and its role in the heart under pathological conditions, including hyperglycemia/diabetes-associated cardiac dysfunction. Additionally, all findings can provide new targets such as cellular [Zn^2+^]_i_-regulation via mediation of Zn^2+^-transporters and suggest that modulation of some endogenous kinases such as CK2α may provide a novel means to correct cardiac dysfunction under any pathological condition.

## 7. Concluding Remarks

**Figure 4:**
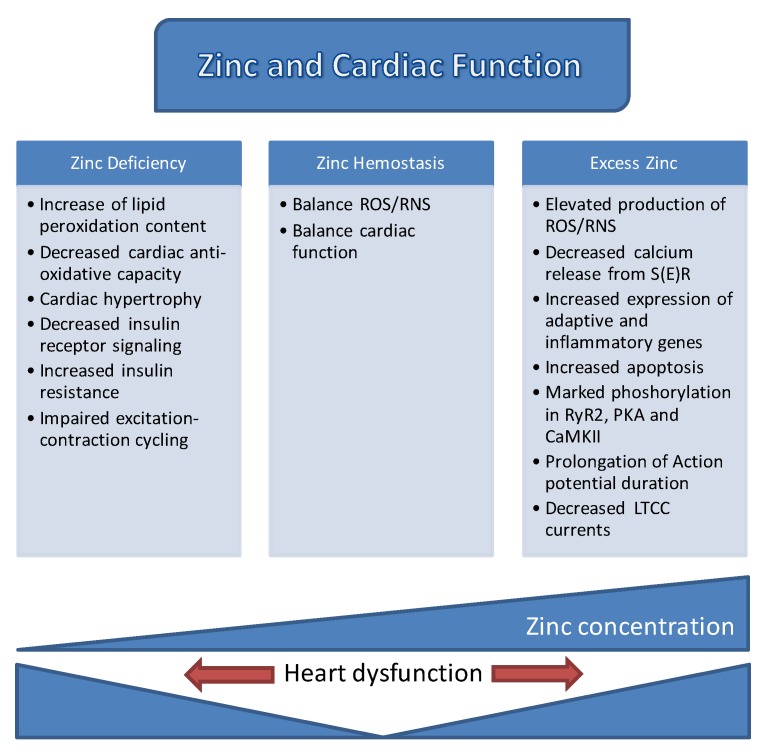
A summary of zinc and its role in cardiac function. Basic mechanisms affected with either zinc-deficiency or zinc-excess and, the, in turn, underline the heart dysfunction, mainly as a zinc-concentration dependent manner in the heart.

Both early and recent studies strongly emphasized how [Zn^2+^]_i_ homeostasis is tightly controlled by the coordinated regulation of its uptake, efflux, distribution, and storage in mammalian cells. A number of proteins involved in different signaling pathways, mitochondrial metabolism, and ion channels, which are also common targets of labile Zn^2+^, play pivotal roles in controlling cardiac contractility. The already known documents associated with the role of zinc in cardiac function are summarized in Figure 4. However, these regulatory actions of Zn^2+^ are not limited to the function of the heart, but also extend to numerous other organ systems in mammalians. In this review, the regulation of cellular labile Zn^2+^ levels, Zn^2+^-mediated signal transduction, impacts of Zn^2+^ on ionic channels and S(E)R, and finally, the roles of Zn^2+^ transporters in healthy and diseased heart, including diabetic heart, were outlined to help widen the current understanding of the versatile and complex roles of Zn^2+^. Although much has been learned from recent studies, revealed important relationships between Zn^2+^ transporters and heart diseases and indicating the potential of Zn^2+^ transporters as therapeutic targets, their precise physiological functions are not clear. Given the multiple roles of Zn^2+^ in various cell types and the detailed research development of Zn^2+^-containing new markers/sensors will improve the ways to handle heart failure in humans.

## Figures and Tables

**Table 1:** Effect of excess Zn^2+^ on cardiomyocyte electrical and mechanical activity.

Parameters	Excess Zn^2+^	Parameters	Excess Zn^2+^
*Electrical activity*		*Mechanical activity*	
Resting membrane potential	**↔**	Muscle Contraction	↓
Action potential duration (APD) Time to peak AP amplitude (TP)	**↔** **↑**	Contraction rate Relaxation rate	↓ ↓
Ca^2+^ transients	**↓**	Time to peak contraction	↔
L-type Ca^2+^ currents	**↓**	Time at 50% of relaxation	↔
Mitochondrial membrane potential	**↑**		

Arrows represent the increased (↑) decreased (↓) or unchanged (↔) electrical and mechanical activity as time or amplitude of the parameters.

**Table 2:** Effects of excess Zn^2+^ on biochemical and ultrastructural parameters in cardiomycoytes.

Parameters	Excess Zn^2+^		Parameters	Excess Zn^2+^
*Biochemical parameters*				
pRyR2/RyR2	↑	Promyelocytic leukemia(PML)		↑
pPKA/PKA	↑	Bcl-2/BAX		↓
FK506-binding protein(FKBP12.6)	↔	pAkt/Akt		↑
pCaMKII/CaMKII	↑	pNFκB/NFκB		↑
Calregulin	↑	pGSK/GSK		↑
Glucose regulated protein (GRP78)	↑			
*Ultrastructure parameters*				
Morphological changes in mitochondria	↑	Electron density of Z-lines		↓
Number of lysosomes	↑			

Arrows represent the increased (↑), decreased (↓), or unchanged (↔) protein expression levels in biochemical parameters or number of lysosomes, Z-lines, and morphological changes in ultrastructure parameters.
